# A potential tool for predicting epidemic trends and outbreaks of scrub typhus based on Internet search big data analysis in Yunnan Province, China

**DOI:** 10.3389/fpubh.2022.1004462

**Published:** 2022-12-01

**Authors:** Zixu Wang, Wenyi Zhang, Nianhong Lu, Ruichen Lv, Junhu Wang, Changqiang Zhu, Lele Ai, Yingqing Mao, Weilong Tan, Yong Qi

**Affiliations:** ^1^Huadong Research Institute for Medicine and Biotechniques, Nanjing, China; ^2^Bengbu Medical College, Bengbu, China; ^3^Chinese PLA Center for Disease Control and Prevention, Beijing, China; ^4^Nanjing Bioengineering (Gene) Technology Center for Medicines, Nanjing, China

**Keywords:** scrub typhus, *Orientia tsutsugamushi*, Baidu Search Index, Internet search big data, autoregressive integrated moving average, ARIMA, ARIMAX

## Abstract

**Introduction:**

Scrub typhus, caused by *Orientia tsutsugamushi*, is a neglected tropical disease. The southern part of China is considered an important epidemic and conserved area of scrub typhus. Although a surveillance system has been established, the surveillance of scrub typhus is typically delayed or incomplete and cannot predict trends in morbidity. Internet search data intuitively expose the public's attention to certain diseases when used in the public health area, thus reflecting the prevalence of the diseases.

**Methods:**

In this study, based on the Internet search big data and historical scrub typhus incidence data in Yunnan Province of China, the autoregressive integrated moving average (ARIMA) model and ARIMA with external variables (ARIMAX) model were constructed and compared to predict the scrub typhus incidence.

**Results:**

The results showed that the ARIMAX model produced a better outcome than the ARIMA model evaluated by various indexes and comparisons with the actual data.

**Conclusions:**

The study demonstrates that Internet search big data can enhance the traditional surveillance system in monitoring and predicting the prevalence of scrub typhus and provides a potential tool for monitoring epidemic trends of scrub typhus and early warning of its outbreaks.

## Introduction

Scrub typhus is an acute febrile illness caused by *Orientia tsutsugamushi*. It is transmitted through chigger bites and is endemic to a “tsutsugamushi triangle” region, covering one billion people at risk and causing one million cases annually (1–3). The case fatality rate was as high as 60% in the pre-antibiotic era (4). Although nowadays antibiotics are available and treatment for scrub typhus has significantly improved, this neglected tropical disease still causes fatal organ failure and deaths due to misdiagnosis and the resultant delay in treatment (5).

China is an important epidemic area included in the “tsutsugamushi triangle” region. In particular, the southern part of the country, located in the tropical or subtropical zone with a hot and humid climate and broad lush vegetation, bears a great burden of scrub typhus disease as the conserved area of *Orientia tsutsugamushi* (6). Typically, hundreds of thousands of cases are reported each month in each province in this area, not to mention the undocumented, underdiagnosed, and misdiagnosed cases, causing a major concern to public health.

In China, a surveillance system for various concerned diseases (including scrub typhus) has been built to monitor the cases and deaths so as to assess the burden of the diseases in the country (7). However, the surveillance system cannot provide real-time monitoring data or predict morbidity trends in scrub typhus. Also, the number of reported human scrub typhus cases is probably only a fraction of the actual cases. Therefore, a more pragmatic approach is needed to monitor and predict the prevalence of scrub typhus in humans in this area.

In recent years, Internet search has become a main way for users to actively obtain information. Every search request of people using search engines reflects their motivation. Big data generated by search intuitively expose the enthusiasm of the public or attention to certain keywords. Instead of the Google search engine, people of China mainly use the Baidu search engine (www.baidu.com), and its publicly available search big data is known as Baidu Search Index (BSI) (7). When search data are used in the public health sector, the user behavior hidden behind these data can reflect the prevalence of diseases (8). Previously, the Internet search big data has been used for monitoring the epidemic trends and early warning of outbreaks of COVID-19; human brucellosis; hand, foot, and mouth disease (HFMD); dengue fever; pertussis; seasonal influenza; etc. (7, 9–16). From these previous studies, it is expected that a new method based on Internet search big data can serve as a potential tool for monitoring epidemic trends and early warning of scrub typhus outbreaks.

Yunnan Province, located in the southwest of China, is one of the provinces that report the highest prevalence of blood-sucking mites, mite-borne agents, and scrub typhus cases in China (6). In the present study, to predict the epidemic trends and outbreak of scrub typhus, a novel prediction model based on the BSI and prevalence data from January 2012 to December 2020 in Yunnan Province was established and evaluated.

## Materials and methods

### Data collection

The number of monthly cases of human scrub typhus in Yunnan Province, China, was obtained from the official website of the Public Health Science Data Center (https://www.phsciencedata.cn/Share/). According to the previously described diagnostic criteria of hospitals in China (17), scrub typhus-positive cases are confirmed when three of the following criteria were present: (1) an outdoor activity history in the epidemic season and area; (2) a high fever with a specific ulcer or eschar; (3) enlargement of the lymph node, liver, and spleen, and presence of rash; and (4) an OXK titer of ≥1:160 or an increase in the OXK titer by ≥4-fold in the Weil–Felix test. The data reported from January 2012 to December 2020 were used for model training and development, and the remaining data from January 2021 to June 2021 were used for external validity assessment.

The monthly BSI data for different keywords, which represented the monthly search engine query data generated by local populations, were obtained from the Baidu Index website (https://index.baidu.com). The BSI data were from January 2012 to June 2021.

Both monthly incidence data and BSI data in an XLSX format using Microsoft Excel software (Microsoft (China) Co., Ltd.) were exported to Python 3.7 software (www.python.org) for subsequent statistical analysis and modeling.

### Model development and evaluation

**Figure 1:**
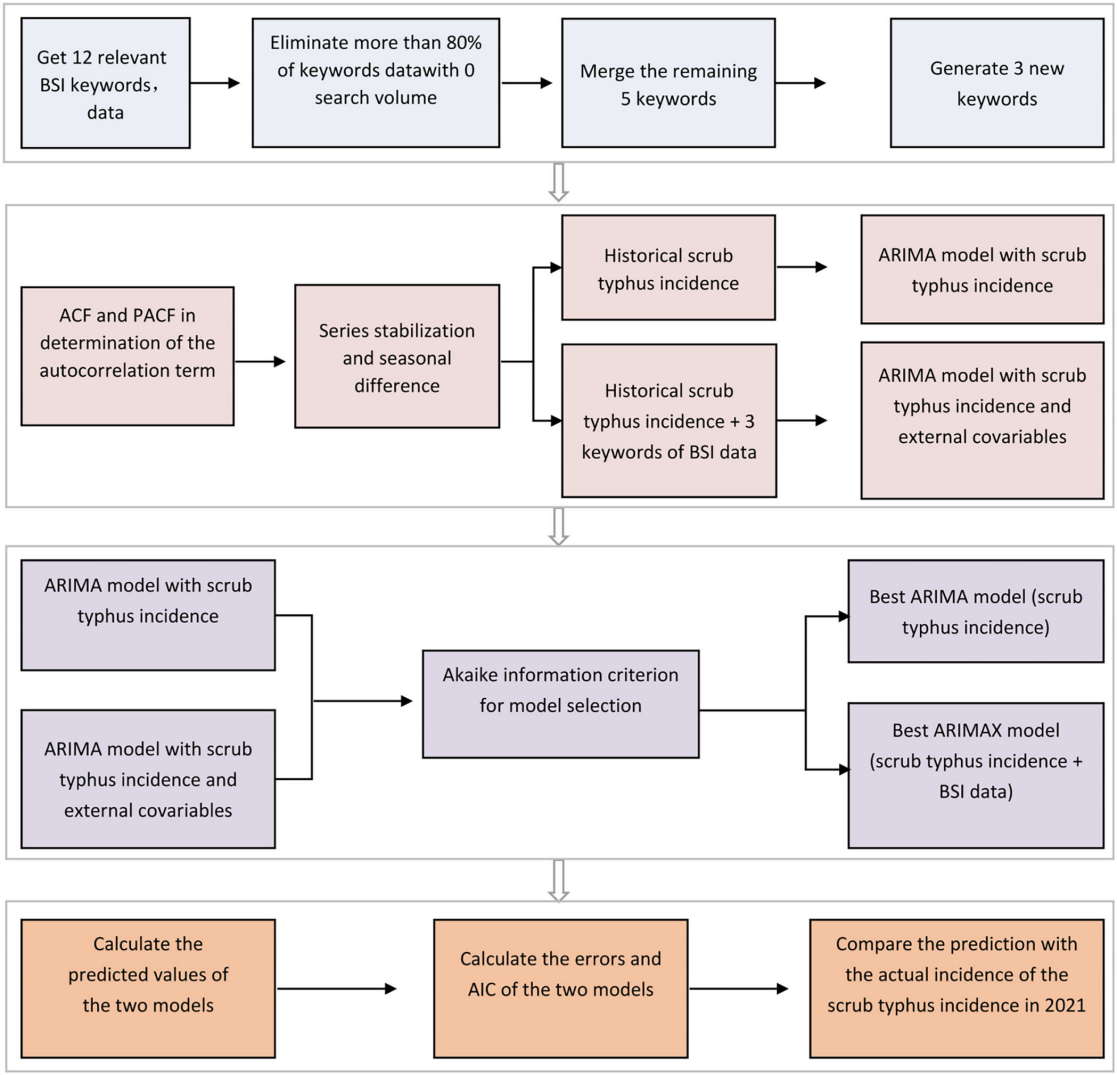
Modeling process in the present study.

The procedures of model development and assessment are indicated in Figure 1. First, 12 keywords related to scrub typhus were used for screening the most relevant results. The correlations and cross-correlations between the BSI data generated from different keywords and the scrub typhus cases were statistically analyzed using Spearman rank correlation and cross-correlation analyses, respectively. The BSI data generated from the keyword or keyword combinations with the most relevant result were used for further model construction.

Second, both the commonly used time series models autoregressive integrated moving average (ARIMA) model and its variant ARIMAX model were used to develop prediction tools, and a comparation was made between them. In the ARIMA model, historical incidence data were used as the only variable to predict the incidence, while in the ARIMAX model, the BSI data were introduced as an external variable in addition to the incidence data for prediction. Also, autocorrelation of the number of scrub typhus cases was determined by the autocorrelation function (ACF) and partial autocorrelation function (PACF). A unit root test, augmented Dickey–Fuller test, was used to determine the necessity of differencing of a time series, and a *P*-value of < 0.05 suggested that differencing was required (7).

Finally, the model showing the best performance was selected based on several criteria, as described previously (7). In brief, a better model should have residuals not distinguished from white noise sequences (analyzed using the Ljung–box test) and the lower absolute value of the Akaike information criterion (AIC) and root-mean-square error (RMSE). In addition, more measurements including mean absolute error (MAE), mean square error (MSE), root-mean-square error (RMSE), mean absolute percentage error (MAPE), and symmetric mean absolute percentage error (SMAPE) were used to evaluate the ARIMA and ARIMAX models to determine the best performing model. Each measurement method has its advantages. MAE can avoid the problem that errors cancel each other out, so it can accurately reflect the size of the actual prediction error. MSE is the averaged sum of the square of the difference between the real value and the predicted value. RMSE is the ratio of the square of the deviation between the observed value and the true value and the number of observations. MAPE is often used to compare multiple time series and is more sensitive to outliers. SMAPE is a revision MAPE. To evaluate the prediction performance of the selected model, the predicted data and actual data of scrub typhus cases from January to June 2021 were compared.

### Statistical tests

All statistical and modeling processes for time series were analyzed using Python 3.7 software in which the “statsmodels” package was used to construct and analyze ARIMA and ARIMAX models. A *P*-value of < 0.05 was considered significant in the analysis.

## Results

### BSI keyword selection

A total of 12 related keywords in Chinese were chosen to obtain the BSI data from January 2012 to June 2021. However, seven keywords showed few BSI data, indicating they were not typically used for search. Of the remaining five keywords (““恙虫” (informal expression form of chigger mite), “羌虫” (another informal expression form of chigger mite), “恙虫病” (meaning scrub typhus), “羌虫病” (another expression of scrub typhus), and “恙螨” (formal expression of chigger mite, K3), the BSI data of “恙虫” and “羌虫” (K2) or “恙虫病” and “羌虫病” (K1) were combined, for they shared the same meaning, though were written differently in Chinese. Finally, three keywords or keyword combinations were obtained in the present study.

### Scrub typhus cases and BSI data

**Figure 2:**
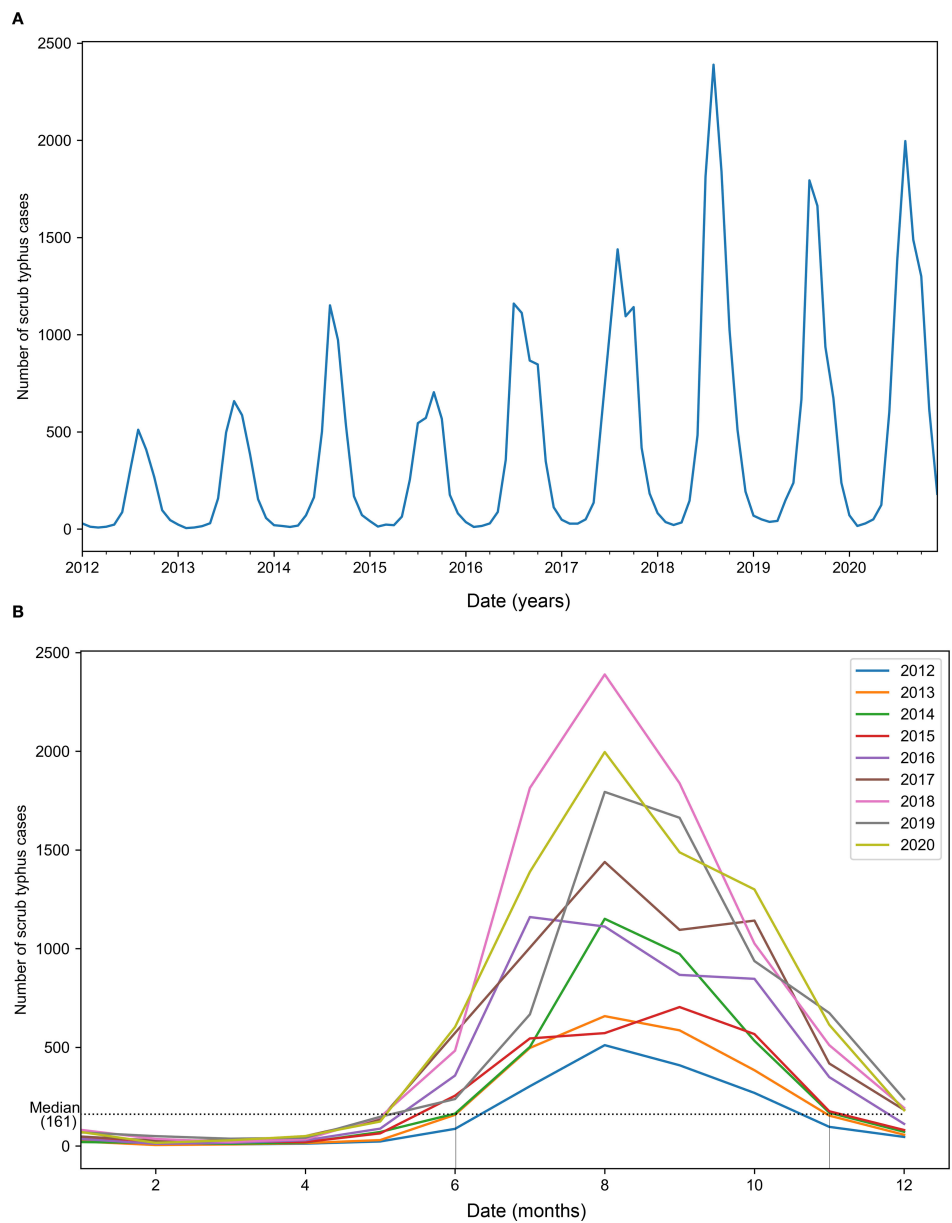
Trends of the actual number of scrub typhus cases from January 2012 to December 2020. **(A)** Overall trend of the number of cases. **(B)** Monthly trend of the number of cases.

From January 2012 to December 2020, there were 45,262 new cases of scrub typhus in Yunnan Province, with an average of 5029.11 cases per year. In general, the number of cases shows an upward trend, with anomalous peaks in 2014 and 2018 (Figure 2A). The disease mainly occurred from June to October (summer and autumn) each year, with strong seasonality of temporal distribution (Figure 2B).

**Figure 3:**
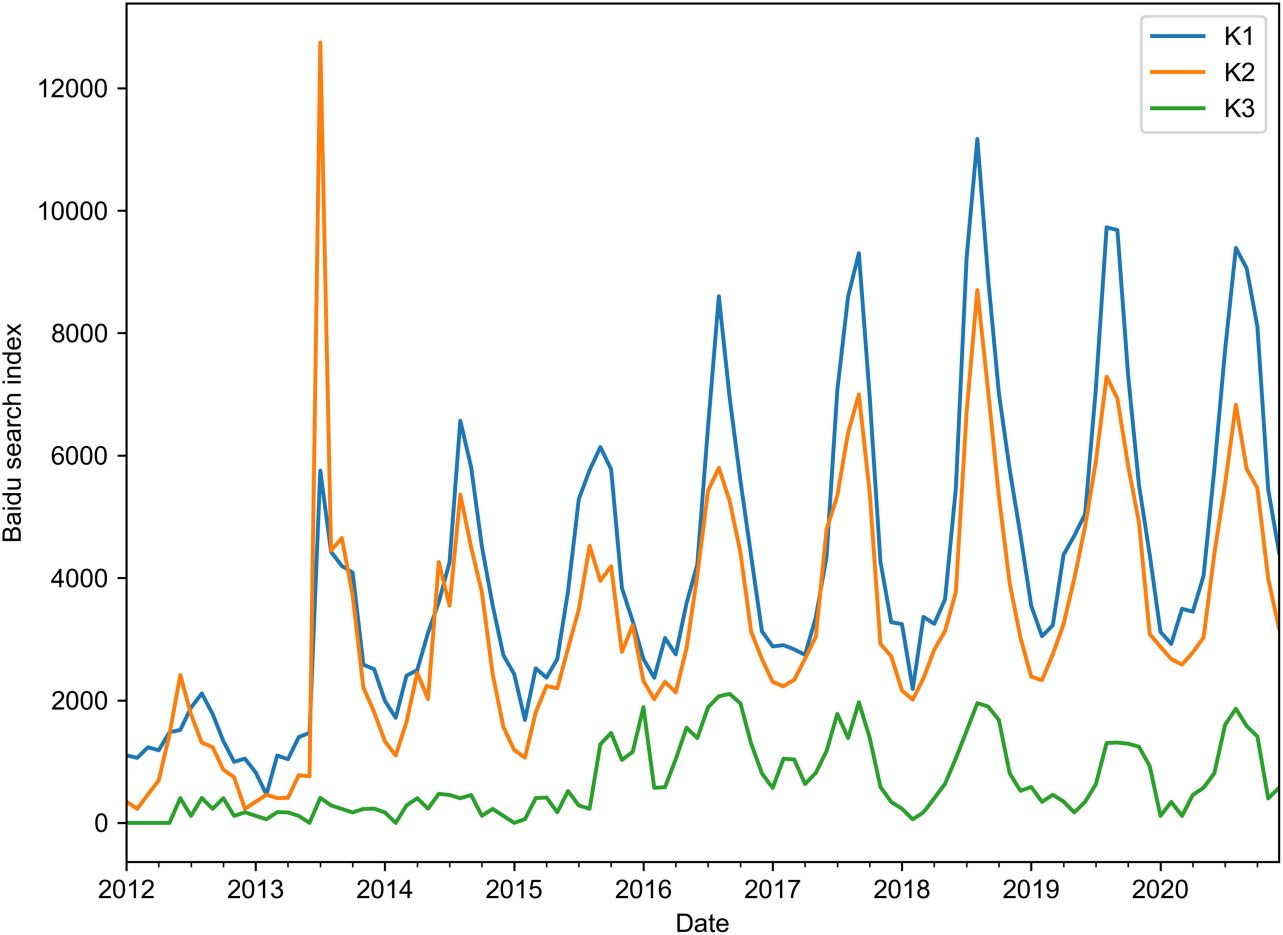
Trends of the Baidu Search Index (BSI) data of three keywords from January 2012 to December 2020.

Unsurprisingly, the BSI data of the selected keywords showed seasonal fluctuations, and the peaks emerged at a similar time to the actual scrub typhus cases (Figure 3). In general, the BSI data of keyword K1 showed a similar trend to the number of scrub typhus cases. However, the BSI data of keyword K2 showed an unusual peak in 2013, and keyword K3 showed relatively less search data than the others, especially before June 2015 (Table 1 and Figure 3).

**Table 1:** Baidu Search Index of the selected keywords.

**Keywords**	**Min**	**Mean**	**Median**	**Max**	**SD**
K1	462	4164	3550	11171	2412
K2	230	3288	2851	12747	2070
K3	0	685	456	2107	607

SD, standard deviation.

Spearman correlation analysis revealed significant positive correlations between the BSI data of each keyword and the number of scrub typhus cases (*P* < 0.001, Table 2), indicating BSI data can be used to reflect the epidemic trend of scrub typhus. Of the three keywords, K1 showed the strongest correlation between its BSI data and the cases, with the largest correlation coefficient and smallest *P*-value. Similarly, cross-correlation analysis showed that the BSI data of K1 had the strongest correlation with the number of cases with the largest cross-correlation function (CCF, 0.912) and a lag of 0 (Table 3).

**Table 2:** Spearman correlation analysis between BSI data of individual keywords and the number of scrub typhus cases.

**Keywords**	**Correlation coefficient**	**P-value**
K1	0.86	0.000
K2	0.85	0.000
K3	0.63	0.000

**Table 3:** Results of cross-correlation analysis between BSI data of individual keywords and the number of scrub typhus cases.

**Keywords**	**Maximum CCF**	**Lag**	**P-value**
K1	0.912	0	<0.01
K2	0.791	0	<0.01
K3	0.701	0	<0.01

CCF, cross-correlation function.

### Seasonal ARIMA and ARIMAX model constructions

The univariate seasonal ARIMA model was constructed to predict the number of scrub typhus cases using past data. As the time series data showed trends and seasonality, data conversion processing was required to eliminate the properties and make it stationary. The enhanced Dickey–Fuller (ADF) test indicated that the time series data were unstable with a *P*-value of > 0.05 (Dickey–Fuller = −0.8552, *P* = 0.802). However, after differentiation, the non-stationary time series data were stabilized with a *P*-value of < 0.001 (Dickey–Fuller = −9.882), indicating the trends and seasonality of the time series were eliminated.

After the data conversion, the ACF and PACF were used to estimate the MA and AR terms of the ARIMA model (Supplementary Figure S1). Judged from the lower absolute value of the AIC in several experiments, ARIMA (1,1,1) (0,1,1) was selected and constructed as the prediction model (Table 4). All parameters were significant with a *P*-value of < 0.01. The residual autocorrelation test (Ljung–box test) showed that the residual was not distinguished from white noise sequences (Q^*^ = 1.90, *P* = 0.27), indicating it is an acceptable model.

**Table 4:** Parameters of ARIMA and ARIMAX models.

	**Parameters**	**Coefficient**	**SE**	**Z**	***P*-value**
ARIMA (1,1,1) (0,1,1)	ar.L1	0.5756	0.060	9.613	0.000
	ma.L1	−1.000	0.096	−10.381	0.000
	ma.S.L12	−0.5454	0.059	−9.237	0.000
	sigma2	4.27e+04	2.26e−06	1.89e+10	0.000
ARIMAX (ARIMA with BSI as external variable)	K1	0.019	0.018	9.832	0.000
	ar.L1	0.3861	0.122	3.164	0.002
	ma.L1	−0.9120	0.071	−12.809	0.000
	ma.S.L12	−0.7415	0.092	−8.078	0.000
	sigma2	3e+04	4134.134	7.256	0.000

SE, standard error; Z, z-test statistic.

Then, ARIMAX, the multivariate version of the ARIMA model, was constructed by introducing BSI data as an external variable. All the individual keywords and keyword combinations were tried, and the AIC value, various error indicators, and R-square were comprehensively compared. As a result, the models using K1 or K1 or K3 showed the best performance with a relatively lower AIC value and various error indicators as well as higher R-square (Supplementary Table S1). According to Occam's razor simplicity principle (if the model performance is similar, it is better to choose the most concise model), K1 was selected to construct the more concise model.

### Model comparation

To evaluate and compare the performances of the two models, various evaluation indexes, including MAE, MSE, MAPE, RMSE, SMAPE, and AIC, were calculated (Table 5), and model 2 (ARIMAX) performed better than model 1 (ARIMA).

**Table 5:** Comparison between the ARIMA and ARIMAX models.

**Evaluation index**	**Model 1 (ARIMA)**	**Model 2 (ARIMAX)**
MAE	131.512	98.832
MSE	23202.353	18176.452
RMSE	152.3237	134.820
MAPE	805.223	55.747
SMAPE	114.851	50.454
AIC	1105.653	1079.468
Ljung-Box *P*	0.167598	0.933308

MAE, mean average error; MSE, mean square error; RMSE, root–mean–square error; MAPE, mean absolute percentage error; SMAPE, symmetric mean absolute percentage error; AIC, Akaike information criterion.

### Forecast performance of the models

**Figure 4:**
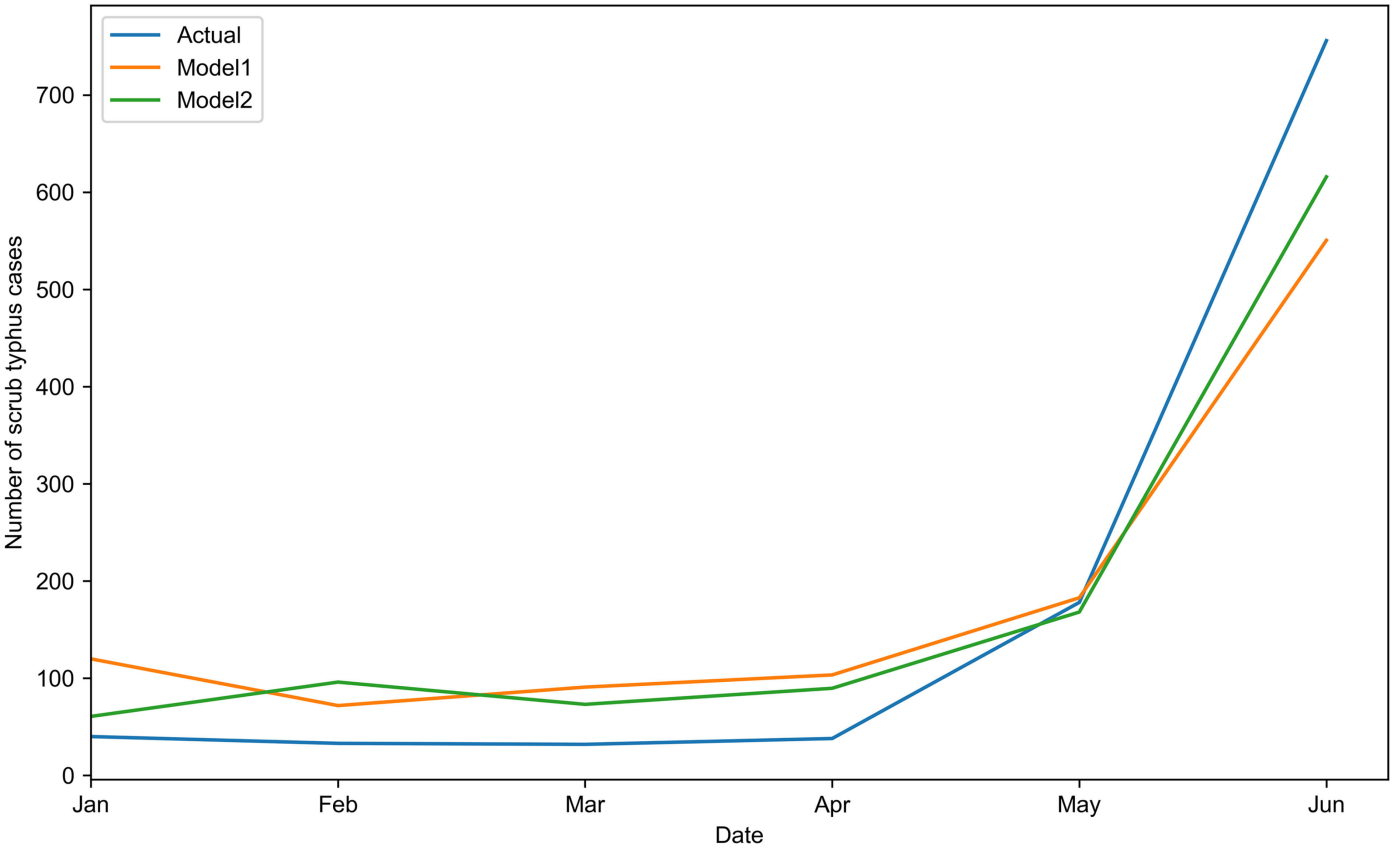
Comparison of the actual scrub typhus cases with the predicted cases by the two models from January to June 2021.

The forecast number of scrub typhus cases from January 2021 to June 2021 was calculated using the two models. The forecast results were compared with the actual number of scrub typhus cases (Table 6 and Figure 4). Based on the prediction performance, model 2 showed a better fitting outcome than model 1, showing lower values of all kinds of error indicators and AIC (Supplementary Table S2).

**Table 6:** Actual and predicted numbers of scrub typhus cases from January to June 2021.

**Date**	**Actual cases**	**Model 1 (ARIMA)**	**Model 2 (ARIMAX)**
		**Predicted cases**	**Predicted cases**
Jan 2021	40	119.960	60.740
Feb 2021	33	71.883	96.034
Mar 2021	32	90.874	73.1364
Apr 2021	38	103.422	89.670
May 2021	178	182.783	168.105
Jun 2021	756	550.577	615.835

## Discussion

Using Internet search big data for monitoring and predicting infectious disease trends has drawn attention (18). To our knowledge, this is the first study that established a prediction model of scrub typhus based on Internet search big data and historical scrub typhus incidence data. Traditional univariate time series models typically use historical data to predict the epidemiological characteristics of diseases. However, our study using a multivariate ARIMAX model gives better prediction results by introducing BSI data as an external variable than the univariate ARIMA model.

For collecting BSI data, selecting an appropriate keyword is of great importance. All kinds of keywords should be considered and carefully excluded. In the present study, some informal (misused or misspelled) but often used Chinese words were also included, considering many susceptible people living in rural areas could easily misspell or misuse these obscure words. Also, BSI data generated from combinations of various keywords might also produce good outcomes. Here, the keyword K1 performed slightly better than K2, as judged by multiple evaluation indexes; however, the combination of K1 and K2 did not show better performance. We suspect that some abnormal BSI data might have affected the overall data stability, for example, the skyrocketing search data of K2 in 2013, as shown in Figure 3. Abnormal BSI data might be due to social hot spots caused by news reports or a sudden public health event of the disease. This is a potential limitation of the models of Internet search big data, and the bias caused by news reports should be adjusted in practical application. Interestingly, the search trend of keyword K1 showed a high correlation with the actual scrub typhus incidence during the indicated period. This phenomenon proved our hypotheses that BSI data—the search data of potential patients or related people—could reflect the actual scrub typhus incidence and help monitor the disease.

There is a limitation to our forecasting tool in predicting the small number of cases. The actual case data were obtained from the national surveillance system, and some external factors such as misdiagnosis or missing reports would impose greater influence on the differences between the actual and forecasting results when the actual case number was small (e.g., < 100 cases), leading to poor consistency between the actual and the forecasting results, as shown in Table 6. However, it is believed that the results are reliable in forecasting a large number of cases and the incidence trends (Table 6 and Figure 4), and further optimization of the model is needed in future to overcome this limitation.

Moreover, the present study demonstrated that BSI data, with advantages of being public, inexpensive, and real-time use, could play an important role in predicting scrub typhus incidence. Importantly, the predicted scrub typhus cases can be calculated immediately and provide supplementary and reference data for public health supervision. The predicted scrub typhus incidence could not only be used for early warning of the disease, thus benefiting early preparation for potential outbreaks, but also be used for assessing the scrub typhus prevention and the control strategy in the investigated area.

Except for the limitations and advantages mentioned earlier, the study currently has only focused on one province of China. Thus, further study should be conducted on a larger area or even the whole country, covering greater latitude and longitude, or a smaller area at the county or town level. In that case, more factors such as uneven distribution of the Internet penetration rate or the Internet search data volume will influence the model effects. As reported by the Internet Society of Yunnan (https://www.ynnet.org.cn/), the number of Internet users in Yunnan Province has reached 31.792 million, with the Internet penetration rate as high as 67.8% by the end of 2021. When the established model is applied to other regions with different Internet penetration rates, its applicability needs to be verified, and parameters need to be adjusted. Nevertheless, the present study provides a basis for future research.

In conclusion, a prediction model for scrub typhus incidence in Yunnan Province, China, was successfully established based on Internet search big data and historical incidence data. The generated prediction data could provide useful information for monitoring, early warning, and prevention, and control strategy assessment of scrub typhus, thus benefiting the prevention and control of the disease.

## Data availability statement

The original contributions presented in the study are included in the article/Supplementary material, further inquiries can be directed to the corresponding authors.

## Author contributions

YQ, ZW, and WT: conceptualization. YQ and ZW: data curation, writing—review, and editing. RL, NL, YM, CZ, LA, and JW: formal analysis. WT and YQ: funding acquisition. WZ and WT: resources. ZW: methodology and software. YQ: writing—original draft. All authors contributed to the article and approved the submitted version.

## Funding

This study was funded by the Medical Science and Technology Projects (BWS20J021, 2022-151, and SYDW_KY[2021]12), Jiangsu Social Development Project (BE2017620), and China National Natural Science Foundation (U1602223).

## Conflict of interest

The authors declare that the research was conducted in the absence of any commercial or financial relationships that could be construed as a potential conflict of interest.

## Publisher's note

All claims expressed in this article are solely those of the authors and do not necessarily represent those of their affiliated organizations, or those of the publisher, the editors and the reviewers. Any product that may be evaluated in this article, or claim that may be made by its manufacturer, is not guaranteed or endorsed by the publisher.

## Supplementary material

Supplementary Figure S1 Autocorrelation function (ACF) and partial autocorrelation function (PACF) analysis of the number of scrub typhus cases.

Supplementary Table S1 All kinds of error indicators and akaike information criterion (AIC) values of the established ARIMAX model with BSI data of various individual keywords or keyword combinations as external variable.

Supplementary Table S2 All kinds of error indicators and akaike information criterion (AIC) values of the two models in prediction of scrub typhus cases in 2021.
