# Supplementary figures and images for: A potential tool for predicting epidemic trends and outbreaks of scrub typhus based on Internet search big data analysis in Yunnan Province, China
Source: Front Public Health. 2022 Dec 1;10:1004462. doi: 10.3389/fpubh.2022.1004462 (PMC9751444; doi:10.3389/fpubh.2022.1004462)

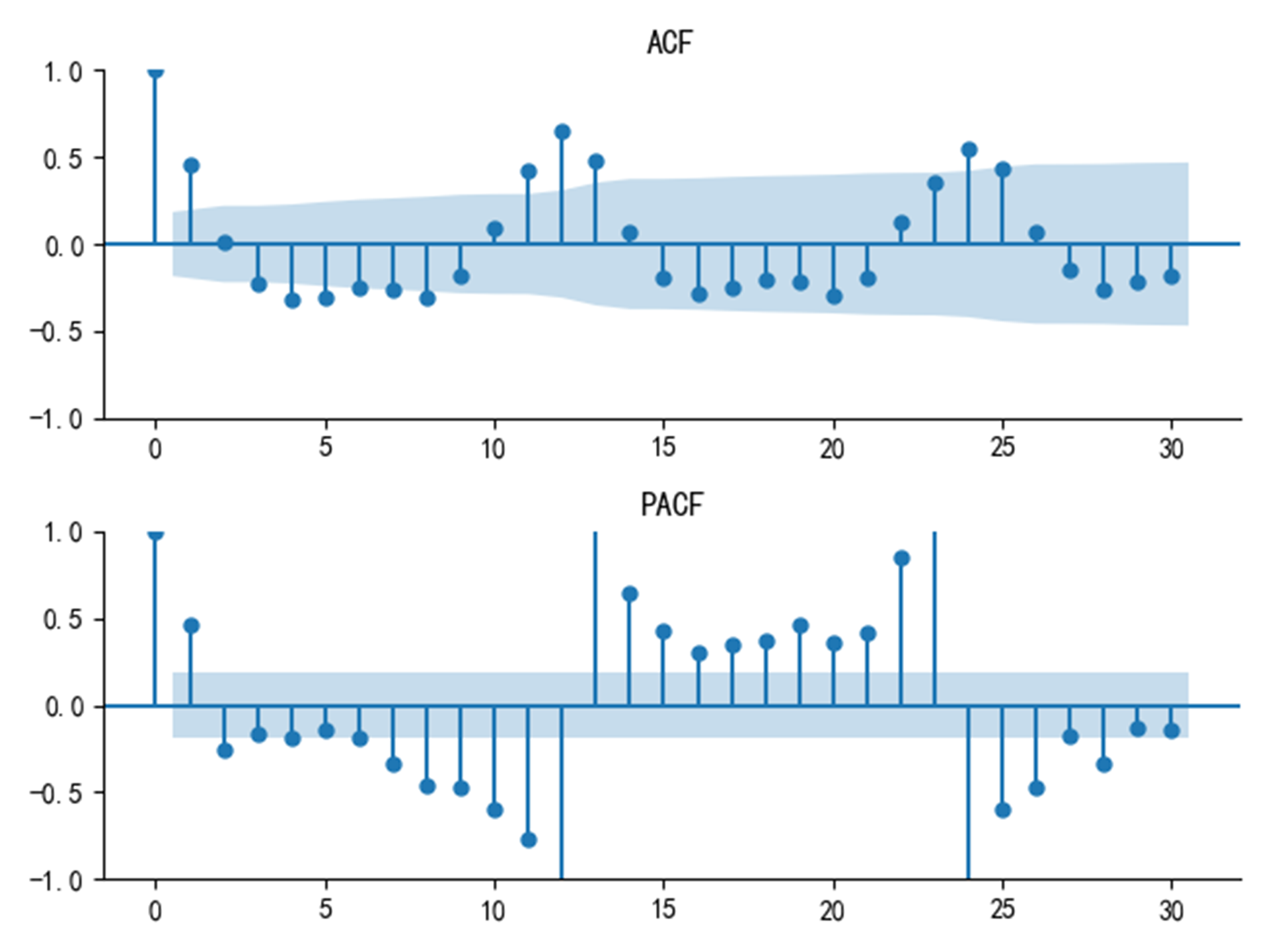

Supplement: Supplementary Figure S1 — Autocorrelation function (ACF) and partial autocorrelation function (PACF) analysis of the number of scrub typhus cases. [file Image_1.TIF]
